# Three New Species of Nolidae (Lepidoptera) from Palawan Island, the Philippines, with Checklists of the Genera *Wittonola*, *Aeneanola*, and *Evonima* †

**DOI:** 10.3390/insects16080775

**Published:** 2025-07-28

**Authors:** Yeong-Bin Cha, Ulziijargal Bayarsaikhan, Jae-Ho Ko, Tak-Gi Lee, Chang-Moon Jang, Hanul Kim, Jeong-Nam Kim, Yang-Seop Bae

**Affiliations:** 1 Division of Forest Biodiversity, Korea National Arboretum, Pocheon 11186, Republic of Korea; cyb0201@naver.com; 2 Convergence Research Center for Insect Vectors, Division of Life Sciences, College of Life Sciences and Bioengineering, Incheon National University, Incheon 22012, Republic of Koreahanul00000@naver.com (H.K.); 3 Division of Life Sciences, College of Life Sciences and Bioengineering, Incheon National University, Incheon 22012, Republic of Korea; rlawjdska167@naver.com; 4 DASARI Research Institute of BioResources, Dunsan-daero, 117 beon-gil, Seo-gu, Daejeon 35203, Republic of Korea; 5 Research Institute of Basic Sciences, Incheon National University, Incheon 22012, Republic of Korea; 6 Diversity Conservation Research Department, Nakdonggang National Institute of Biological Resources, Sangju 37242, Republic of Korea; 7 Bio-Resources and Environmental Center, Incheon National University, Incheon 22012, Republic of Korea

**Keywords:** Oriental region, Distribution, new record, Cleopatra’s needle, Nolinae

## Abstract

This paper contains three newly recorded genera (*Wittonola* László, Ronkay & Ronkay, 2015; *Aeneanola* László, Ronkay & Ronkay, 2013; and *Evonima* Walker, 1865) and each new species (*W. bicyana* **sp. nov.**, *A. crassa* **sp. nov.**, and *E. palawanensis* **sp. nov.**) from Palawan, the Philippines. We provide illustrations of three new species and their genitalia, photographs of collecting sites, and checklists of the three genera.

## Simple Summary

During three field trips to the northern part of Palawan Island, we found three new nolid species. In this study, three genera (*Wittonola* László, Ronkay & Ronkay, 2015; *Aeneanola* László, Ronkay & Ronkay, 2013; and *Evonima* Walker, 1865) are newly reported from Palawan Island, the Philippines; among them, the first two genera are new to the Philippines. As a result of this study, the number of *Wittonola* species increases to 2, *Aeneanola* to 5, and *Evonima* to 19 in the world. In this study, we aim to provide three new adult and genitalia illustrations, collecting sites, and each genus’s checklist.

## 1. Introduction

Cleopatra’s needle is one of the critical habitats of Palawan Island, the Philippines [1]. This area covers 41,350 hectares with approximately 50% being primary forests, and almost 85% of endemic species coexist here [2]. According to Lee et al. [3], only 328 species, including 13 CR, 24 EN, 44 VU species, have been known to inhabit Cleopatra’s needle. Despite their importance for biodiversity, many species of animals (reptiles, small mammals, and insects), as well as numerous plants, need to have their conservation status evaluated [2]. To enhance our understanding of Palawan’s insect fauna, we examined Nolinae specimens collected from the island and discovered a newly recorded genus, *Wittonola* László, Ronkay & Ronkay, 2015, from the Philippines, and two additional genera, *Aeneanola* László, Ronkay & Ronkay, 2013, and *Evonima* Walker, 1865, newly recorded in Palawan, each represented by a new species.

The genus *Wittonola* László, Ronkay & Ronkay, 2015 has been recorded only in Southeast Asia, specifically Cambodia and Indonesia, and currently comprises a single species, *W. latifascia* László, Ronkay & Ronkay, 2015 [4]. This genus is defined by the complete fusion of M3 + CuA1 hindwing venation. In the male genitalia, the only known diagnostic character is a very short, button-like harpe. In the female genitalia, the papillae anales are narrow and elongated with extraordinarily long apophyses and a single, thorn-like signum, all of which serve as diagnostic characters for the genus.

The genus *Aeneanola* László, Ronkay & Ronkay, 2013 was established with the discovery of *Pisara acontioides* Walker, 1862 and initially included three additional species: *A. seria* (Holloway, 2003), *A. kalisi* László, Ronkay & Ronkay, 2013, and *A. joiceyi* László, Ronkay & Ronkay, 2013. This group is distributed from South to Southeast Asia [5]. The external appearance of *Aeneanola* closely resembles that of *Calonola* László, Ronkay & Witt, 2010, but the apex of the forewing is shorter, broader, and rounder than in that genus. Their male genitalia are more clearly characterized as follows: a roughly triangular valva; a long, dentated, acute harpe; and an elongated aedeagus bearing a single robust spinous cornutus. The female genitalia possess a long ductus bursa with a caudal portion strongly sclerotized and a partially sclerotized cervix of corpus bursae.

The genus *Evonima* Walker, 1865 is widely distributed across Eurasia and Africa [6,7]. According to Holloway [8], who followed the concept proposed by Inoue [9], the genus is characterized by a reduced and trifine hindwing venation, a colorful forewing, and ciliated male antennae. In the male abdomen, the eighth tergite bears apodemes, while the corresponding sternite is absent. The male genitalia exhibit a long uncus accompanied by a similarly narrow tegumen, and two linear bands are present on the subscaphium. The valva is rectangle-like and bears a blunt harpe arising obliquely toward the ventral margin, often with a few small knobs. The aedeagus is straight and lacks any process. These characters are relatively stable across the genus. The female genitalia are characterized by short, triangle-like ovipositor lobes, a long and slender ductus bursae, and a pyriform corpus bursae bearing a single signum, which is represented by an elongated, narrow, scobinated plate. For more detailed diagnostics, see Holloway [8] and László [6]. A total of 18 species of *Evonima* have been reported to date, distributed across the African, Oriental, and Palearctic regions [6,7,8,9,10,11,12,13,14].

As a result of this study, *Wittonola* is now represented by 2 species (*W. latifascia*, *W. bicyana* **sp. nov.**), *Aeneanola* by 5 species (*A. acontioides*, *A. seria*, *A. kalisi*, *A. joiceyi*, and *A. crassa* **sp. nov.**), and *Evonima* by 19 species in total, including 11 species from the Oriental region (*E. albifurca*, *E. aperta*, *E. faircloughi*, *E. kamboranga*, *E. maculata*, *E. minora*, *E. ochritincta*, *E. plagiola*, *E. unicolor*, *E. xanthoplaga*, and *E. palawanensis* **sp. nov.**).

## 2. Materials and Methods

The examined specimens are preserved in the private collection of the first author (Phil). The specimens were captured using tent light sheets illuminated by a mercury vapor lamp (220V/200W; Dongseong Co., Namyangju, Republic of Korea) and four blacklight lamps (18W; FL20SBL; Kumho Co., Seoul, Republic of Korea).

Images of INU adults were taken by a Tucsen Dhyana 400DC digital camera (Fuzhou, China) attached to a Leica S6D stereomicroscope, with dome illuminator Leica LED5000 HDI. Genitalia were dissected and examined under a Leica S9E stereomicroscope or Leica EZ4 stereomicroscope. Images of genitalia were taken using a Tucsen Dhyana 400DC digital camera mounted on a Leica Z16 APO stereomicroscope (Wetzlar, Germany).

## 3. Results

### 3.1. Taxonomic Accounts

#### 3.1.1. The New Species of Genus *Wittonola* László, Ronkay & Ronkay, 2015 with a Checklist

Superfamily Noctuoidea Latreille, 1809

Family Nolidae Bruand, 1846

Subfamily Nolinae Hampson, 1894

**Genus *Wittonola* László, Ronkay & Ronkay, 2015** [4]: 278. Type species: *Wittonola latifasciata* László, Ronkay & Ronkay, 2015 by monotypy.

 

**Figure 1:**
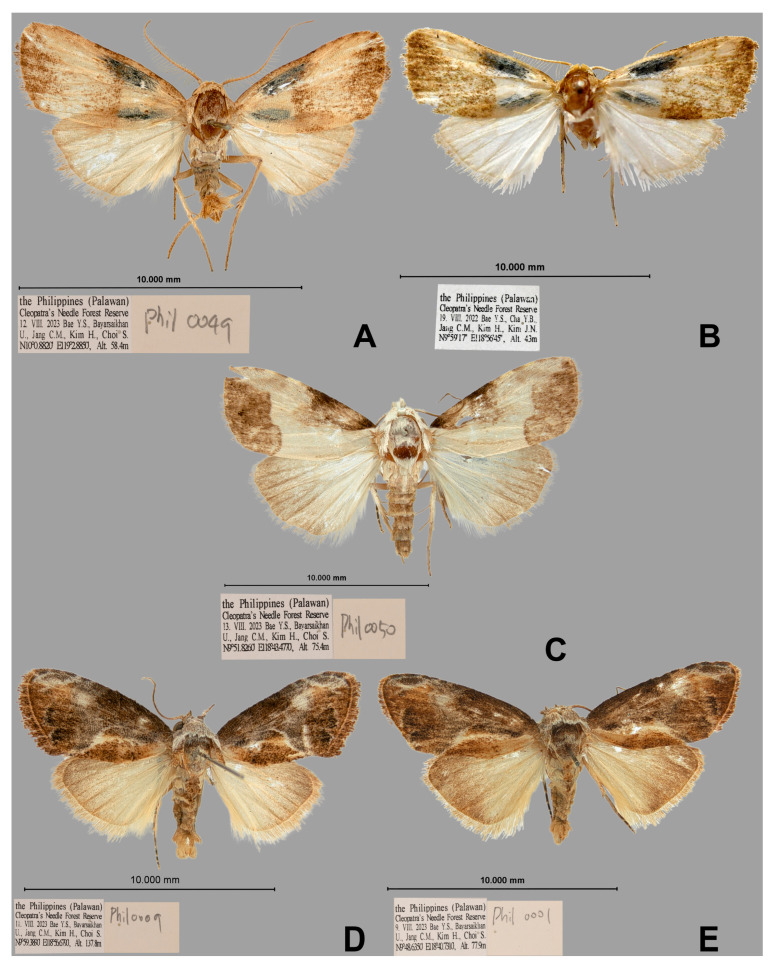
**Three new species of male adults from Palawan, the Philippines.** (**A**) *Wittonola bicyana* **sp. nov.**, **Holotype**, Phil-0049. (**B**) *ditto*, **Paratype**. (**C**). *Aeneanola crassa* **sp. nov.**, **Holotype**, Phil-0050. (**D**) *Evonima palawanensis* **sp. nov.**, **Holotype**, Phil-0009. (**E**) *ditto*, **Paratype**, Phil-0001. (Scale bar on each figure).

**Figure 2:**
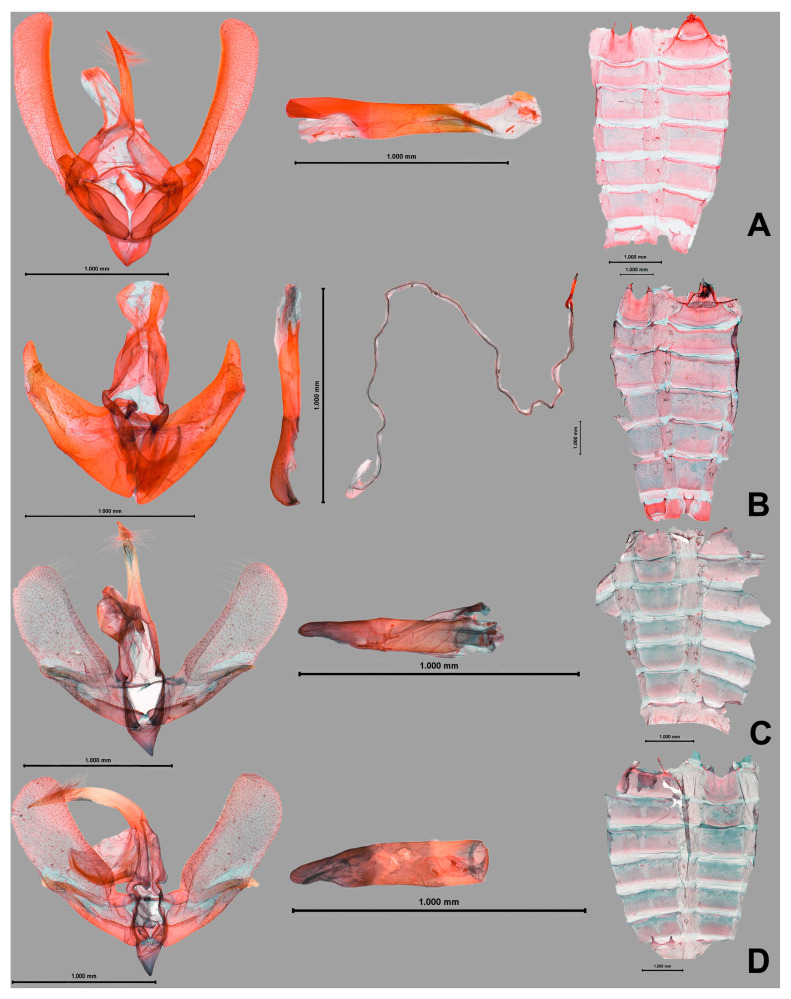
**The male genitalia of new species from the Philippines.** (**A**) *Wittonola bicyana* Cha **sp. nov.**, **Holotype**, Phil-0049. (**B**) *Aeneanola crassa* Cha **sp. nov.**, **Holotype**, Phil-0050. (**C**) *Evonima palawanensis* Cha **sp. nov.**, **Holotype**, Phil-0009. (**D**) *ditto*, **Paratype**, Phil-0010. (Scale bar on each figure).

**Figure 3:**
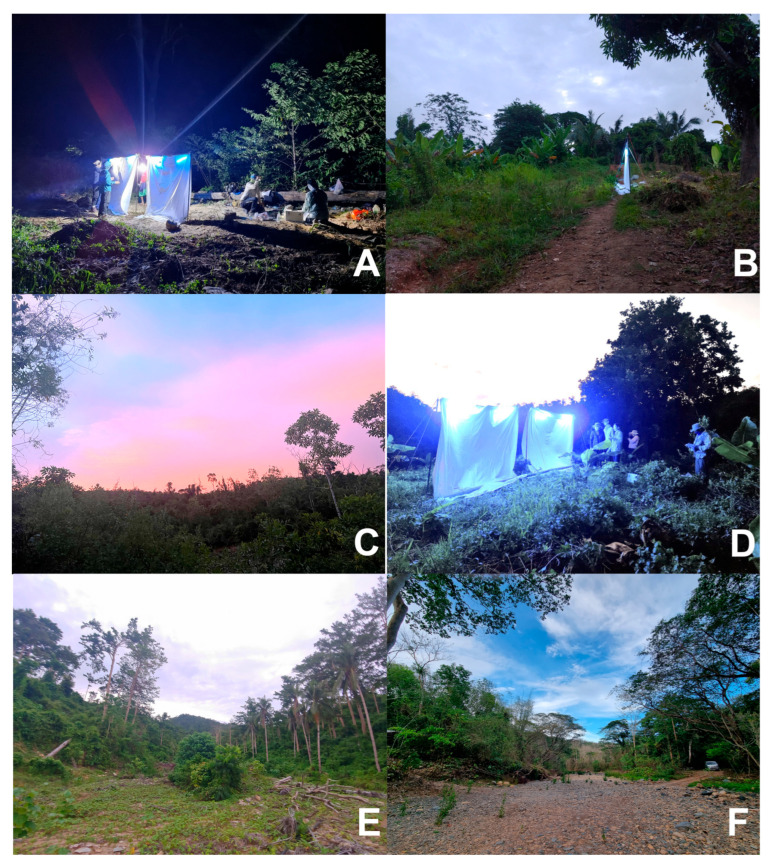
**Habitats of new species from Palawan.** (**A**) Cleopatra’s Needle Forest Reserve (12. VIII. 2023). (**B**) Cleopatra’s Needle Forest Reserve (19. VIII. 2022). (**C**) Cleopatra’s Needle Forest Reserve (13. VIII. 2023). (**D**) Cleopatra’s Needle Forest Reserve (11. VIII. 2023). (**E**) Cleopatra’s Needle Forest Reserve (20. VIII. 2022). (**F**) Cleopatra’s Needle Forest Reserve, near areas inhabited by indigenous peoples (12. V. 2024).

*Wittonola bicyana* Cha **sp. nov.**

(Figure 1A,B, Figure 2A and Figure 3A)

 

**Holotype.** m#, the Philippines (Palawan), Cleopatra’s Needle Forest Reserve (N10°0.8820′, E119°2.8850′, Alt.: 58.4 m) 12. VIII. 2023. (Y.S. Bae, U. Bayarsaikhan, C.M. Jang, H. Kim, S. Choi), genitalia slide No. Phil-0049. **Paratype.** m#, the Philippines (Palawan), Cleopatra’s Needle Forest Reserve (N9°59′17″, E18°56′45″, Alt.: 43 m) 19. VIII. 2022. (Y.S. Bae, Y.B. Cha, C.M. Jang, H. Kim, J.N. Kim), abdomen loss.

**Diagnosis.** The only comparable species is *W. latifasciata* László, Ronkay & Ronkay, 2015. The new species’ habitus is quite distinct from its congener. The medial band is very faint, with two prominent dark blue streaks located near the costal and dorsal areas of the antemedial region. Additionally, its hindwing is paler compared to that of *W. latifasciata*. In male genitalia, *W. bicyana* **sp. nov.** possesses a more porrect valva and a more elongated harpe. In the aedeagus, two striking features are evident: the well-developed carina processes and a sclerite-like cornutus with a granulated vesica.

**Description.** *Adult* (Figure 1A,B). Antenna bipectinate in males. Head and thorax pale ocher. Forewing length: 8 mm in males (wingspan: 15 mm). Ground color of forewing: pale ocher. Two dark blue streaks on antemedio-subcostal and subdorsal. Dark brown medial band inconspicuous, inner line porrect but outer line wavy. Dark brown area covered subterminal to terminal, except apical area. Ground color of hindwing: pale gray, gradual darkening to terminal. Abdomen pale brownish gray.

*Male genitalia* (Figure 2A). Uncus half-length of valva, tapering, with linearly sclerotized subscaphium. Tegumen wide. Transtilla thin, sclerotized. Valva elongated; costal margin well-developed, arched. Harpe molar-shaped, somewhat arising from valva. Sacculus moderate. Juxta broadened V-shape. Vinculum V-shaped. Aedeagus porrect; coecum long as dorsal carina process. Two carina processes present, dorsal process longer than ventral process, and downward. A sclerite-like cornutus with granulated vesica. The 8th tergite is weakly sclerotized, Y-shaped anteriorly; 8th sternite anterior margin is weakly sclerotized.

**Distribution.** The Philippines (Palawan).

**Remark.** This genus has originally only been reported to be from Cambodia and Indonesia (Sumatra) [4]. Here, we firstly report outside of its type localities, and this is the first record from the Philippines.

**Etymology.** The specific epithet *bicyana* is a compound adjective formed from the Latin prefix bi-, meaning “two,” and cyana, derived from the Latin adjective *cyaneus* (in its feminine form), meaning “deep blue.” It refers to the paired deep blue character [or coloration] of the species and is used adjectivally in agreement with the feminine genus *Wittonola*.

**Habitat.** Type series collection sites are illustrated (Figure 3A,B).

 

**Checklist of the genus *Wittonola* László, Ronkay & Ronkay, 2015 of the world**

1. ***Wittonola bicyana* Cha sp. nov.**
  *Distribution.* The Philippines (Palawan).
2. *Wittonola latifasciata* László, Ronkay & Ronkay, 2015
  *Wittonola latifasciata* László, Ronkay & Ronkay, 2015: 279. Type locality: Cambodia: Mondolkiri: Seima Biodiversity Conservation Area, between Seima and O’Rang, holotype: female, in coll. MWM.
  *Distributions*. Cambodia, Indonesia (Sumatra) [4].

#### 3.1.2. The New Species of Genus *Aeneanola* László, Ronkay & Ronkay, 2015 with a Checklist

**Genus *Aeneanola*** László, Ronkay & Ronkay, 2013 [5]: 172. Type species: *Pisara acontioides* Walker, 1862.

 

*Aeneanola crassa* Cha **sp. nov.**

(Figure 1C and Figure 2B)

 

**Holotype.** m#, the Philippines (Palawan), Cleopatra’s Needle Forest Reserve (N9°51.8260′, E118°43.4770′, Alt.: 75.4 m) 13. VIII. 2023. (Y.S. Bae, U. Bayarsaikhan, C.M. Jang, H. Kim, S. Choi), genitalia slide No. Phil-0050.

**Diagnosis.** This species resembles *Calonola* László, Ronkay & Ronkay, 2015 spp., due to the underdeveloped metallic characteristics of *Aeneanola*. However, its genitalia structure clearly aligns with the genus *Aeneanola*. The new species can be distinguished by the aforementioned characteristics, though the genitalia provide more precise differentiation.

In male genitalia, *A. seria* (Holloway) remains unknown; *A. acontioides* (Walker) exhibits a long valva and harpe; *A. kallisi* László, Ronkay & Ronkay, 2013 has a shorter valva and harpe compared to *A. acontioides*; and *A. joiceyi* László, Ronkay & Ronkay, 2013 presents the shortest valva and harpe among these species. In contrast, *A. crassa* Cha **sp. nov.** is characterized by an arched valva and a distinctly curved, claw-like harpe. Additionally, the aedeagus of *A. crassa* **sp. nov.** is relatively long and porrect compared to other species.

**Description.** *Adult* (Figure 1C). Antenna bipectinate in male. Head and thorax creamy. Forewing length: 7 mm (wingspan 15 mm) in males. Ground color of forewing creamy. Black hemi-circular patch with interrupted faint cream medially from baso- to medio-costa. Stair-like distal huge patch, dim dark brown with imperceptible scale on subterminal line. Cilia dim dark brown. Ground color of hindwing creamy, darkening toward terminal. Abdomen anteriorly creamy, posteriorly dim, dark brown.

*Male genitalia* (Figure 2B). Uncus stout, apically bifurcate weakly, with linearly sclerotized subscaphium. Tegumen moderate. Transtilla sclerotized. Valva saber-shaped; costal margin well-sclerotized; harpe basally round triangle, crest-like plate with a curved claw-shaped process. Sacculus well-sclerotized. Vinculum V-shaped. Aedeagus weakly arched near coecum with a small spinous cornutus; ductus ejaculatorious rather long. The 8th tergite Y-like sclerite with two thin processes anteriorly; 8th sternite well-sclerotized without posterior triangle area, with two small horn-like processes anteriorly.

**Distribution.** The Philippines (Palawan).

**Remark.** This genus has been reported from the Philippines for the first time.

**Etymology.** The specific epithet *crassa* is a Latin adjective meaning “thick”. It refers to the thick shape of the harpe and is used adjectivally in agreement with the feminine genus *Aeneanola*.

**Habitat.** Holotype collection site is illustrated (Figure 3C).

 

**Checklist of the genus *Aeneanola* László, Ronkay & Ronkay, 2013 of the world**

1. ***Aeneanola acontioides* (Walker, 1862)**
  *Pisara acontioides* Walker, 1862: 118. Type locality: Borneo: Sarawak, holotype: male, in coll. OUMNH.
  *Distributions.* Borneo, Indonesia (Bali, Java, Sumbawa, Sumatra), Nepal, the Philippines, Sri Lanka, Vietnam [5,8,15].
2. ***Aeneanola crassa* Cha sp. nov.**
  *Distribution*. The Philippines (Palawan).
3. ***Aeneanola seria* (Holloway, 2003)**
  *Aquita seria* Holloway, 2003: 20. Type locality: Borneo: Brunei: Seria, holotype: female, in coll. NHMUK.
  *Distribution*. Borneo (Brunei) [8].
4. ***Aeneanola kalisi* László, Ronkay & Ronkay, 2013**
  *Aeneanola kalisi* László, Ronkay & Ronkay, 2013: 173. Type locality: [S.W. Sulawesi] S.W. Celebes: Pangean, near Maros, holotype: male, in coll. NHMUK.
  *Distributions*. Indonesia (Sulawesi, Sumatra) [5].
5. ***Aeneanola joiceyi* László, Ronkay & Ronkay, 2013**
  *Aeneanola joiceyi* László, Ronkay & Ronkay, 2013: 173. Type locality: [Sri Lanka] Ceylon: Polgahawela, holotype: male, in coll. NHMUK.
  *Distribution*. Sri Lanka [5].

#### 3.1.3. The New Species of Genus *Evonima* Walker, 1865 with a Checklist

**Genus *Evonima* Walker, 1865** [16]: 505. Type species: *Evonima aperta* Walker by monotypy.

 

*Evonima palawanensis* Cha **sp. nov.**

(Figure 1D,E and Figure 2C,D)

 

**Type series. Holotype.** m#, the Philippines (Palawan), Cleopatra’s Needle Forest Reserve (N9°59.3890′, E118°56.6790′, Alt.: 137.8 m) 11. VIII. 2023. (Y.S. Bae, U. Bayarsaikhan, C.M. Jang, H. Kim, S. Choi), genitalia slide No. Phil-0009. **Paratypes.** (totally 34m#) 4m# the Philippines (Palawan), Cleopatra’s Needle Forest Reserve (N9°59′35″, E118°57′52″, Alt.: 42 m) 18. VIII. 2022. (Y.S. Bae, Y.B. Cha, C.M. Jang, H. Kim, J.N. Kim); 5m# the Philippines (Palawan), Cleopatra’s Needle Forest Reserve (N9°59′17″, E118°56′45″, Alt.: 43 m) 19. VIII. 2022. (Y.S. Bae, Y.B. Cha, C.M. Jang, H. Kim, J.N. Kim); 11m# the Philippines (Palawan), Cleopatra’s Needle Forest Reserve (N9°59′27″, E118°57′13″, Alt.: 37 m) 20. VIII. 2022. (Y.S. Bae, Y.B. Cha, C.M. Jang, H. Kim, J.N. Kim); m#, the Philippines (Palawan), Cleopatra’s Needle Forest Reserve (N9°48.6350′, E118°40.7310′, Alt.: 77.9 m) 9. VIII. 2023. (Y.S. Bae, U. Bayarsaikhan, C.M. Jang, H. Kim, S. Choi), genitalia slide No. Phil-0001; 2m#, Philippines (Palawan), Cleopatra’s Needle Forest Reserve (N10°0.3310′, E118°58.9930′, Alt.: 53.6 m) 10. VIII. 2023. (Y.S. Bae, U. Bayarsaikhan, C.M. Jang, H. Kim, S. Choi); 2m#, with the same data as in the holotype, genitalia slide Nos. Phil-0010; m#, the Philippines (Palawan), Cleopatra’s Needle Forest Reserve (N10°0.8820′, E119°2.8850′, Alt.: 58.4 m) 12. VIII. 2023. (Y.S. Bae, U. Bayarsaikhan, C.M. Jang, H. Kim, S. Choi); m#, the Philippines (Palawan), Cleopatra’s Needle Forest Reserve (N9°48.6350′, E118°40.7310′, Alt.: 77.9 m) 14. VIII. 2023. (Y.S. Bae, U. Bayarsaikhan, C.M. Jang, H. Kim, S. Choi); 4m#, the Philippines (Palawan), Cleopatra’s Needle Forest Reserve (N9°49.0660′, E118°40.7010′, Alt.: 76.2 m) 10. V. 2024. (Y.S. Bae, U. Bayarsaikhan, H. Kim, J.N. Kim, T.W. Lee), genitalia slide No. Phil-0004; 2m#, the Philippines (Palawan), Cleopatra’s Needle Forest Reserve (N9°49.0660′, E118°40.7010′, Alt.: 76.2 m) 12. V. 2024. (Y.S. Bae, U. Bayarsaikhan, H. Kim, J.N. Kim, T.W. Lee), genitalia slide Nos. Phil-0003, -0005; m#, the Philippines (Palawan), Cleopatra’s Needle Forest Reserve (N10°1.5720′, E119°0.8080′, Alt.: 68 m) 14. V. 2024. (Y.S. Bae, U. Bayarsaikhan, H. Kim, J.N. Kim, T.W. Lee), genitalia slide No. Phil-0002.

**Diagnosis.** This new species is morphologically close to *E. faircloughi* Holloway, 2003. However, key distinguishing features include differences in forewing and male genitalia. The forewing of the new species exhibits a rufous-brown coloration covering 2/3 of the tornal margin and the terminal area, with blackish scales dominating over other markings. In contrast, *E. faircloughi* displays irregular white markings encircling a large ring, whereas the new species shows a faint white line without a ring. Furthermore, the tornal margin in the new species is obtusely rounded, differing from the straight margin of *E. faircloughi*. In male genitalia, the new species is characterized by a more sclerotized subscaphium, larger juxta, downward dentated harpe, and an elongated vinculum. Notably, the absence of the aedeagus in the original description and figure of *E. faircloughi* precludes direct comparison between the two species for this structure.

**Description.** *Adult* (Figure 1D,E). Antenna ciliated in males. Head and patagium creamy white; tegula and thorax rufous brown. Forewing length: 6–7 mm (wingspan: 13–16 mm) in males. Forewing ground color dark gray, almost 2/3 of tornal area and postmedial to terminally rufous brown, except apical area; dark gray area with white margin invades middle of both brown areas like thorn shape. Costal 2/3 area brownish dark gray, with a creamy circular marking between dark gray area and terminal brownish area. Two black half-moon patches with white distal margin on subterminal area. Cilia rufous brown. Ground color of hindwing creamy, darkening toward apex. Abdomen creamy brown.

*Male genitalia* (Figure 2C,D). Uncus long, beak-shaped, with two linearly sclerotized subscaphium. Tegumen moderate. Transtilla thin, weakly sclerotized. Valva elongated; costal margin weakly sclerotized; harpe digitus, apical half dentated ventrally. Sacculus moderate. Juxta wide V-shaped with lateral process. Vinculum V-shaped. Aedeagus moderate without cornutus, but vesica weakly sclerotized; ductus ejaculatorius very long. The 8th tergite with ribbon-like medial plate with gently arched margin, with two stouts, curved processes erecting near both sides of the medial plate; 8th sternite with sclerotized anterior margin.

**Distribution.** The Philippines (Palawan).

**Remark.** Only species of *E. albifurca* (Hampson) has been reported from Luzon Island, the Philippines [7,8,14,17]. Here, we report this genus from Palawan Island for the first time.

**Etymology.** The species name *palawanensis* is derived from the type locality, Palawan Island, the Philippines.

**Habitat.** Type series collection sites are illustrated (Figure 3A,C–F).

 

**Checklist of the genus *Evonima* Walker, 1865 of the world**

1. ***Evonima albifurca* (Hampson, 1914)**
  *Nola albifurca* Hampson, 1914: 411. Type locality: the Philippines: [Luzon]: Manila, holotype: female, in coll. NHMUK.
  *Distribution.* The Philippines [17].
2. ***Evonima aperta* Walker, 1865**
  *Evonima aperta* Walker, 1865: 506. Type locality: [Indonesia]: Java, syntypes: 2 females, in coll. NHMUK.
  *Distributions.* Borneo, China (Taiwan), India (Mizoram), Indonesia (Java, Sumatra), Peninsular Malaysia, Thailand (Chiang Mai, Loei, Nan) [8,10,11,12,16,18,19,20].
3. ***Evonima elegans* Inoue, 1991**
  *Evonima elegans* Inoue, 1991: 75. Type locality: Taiwan: Chiai Hsien: Shihtzulu, holotype: male, in coll. NHMUK.
  *Distributions*. China (Guangdong, Guanxi, Guizhou, Sichuan, Taiwan, Zhejiang) [7,8,9,13,14].
4. ***Evonima faircloughi* Holloway, 2003**
  *Evonima faircloughi* Holloway, 2003: 31. Type locality: [Borneo]: Brunei, holotype: male, in coll. NHMUK.
  *Distribution*. Borneo (Brunei) [8].
5. ***Evonima kamboranga* Holloway, 2003**
  *Evonima kamboranga* Holloway, 2003: 32. Type locality: Borneo, Sabah, Mt. Kinabalu, Kamborangoh; holotype: male, in coll. NHMUK.
  *Distribution*. Borneo (Sabah) [8].
6. ***Evonima lancangensis* Han & Hu, 2019**
  *Evonima lancangensis* Han & Hu, 2019: 445. Type locality: China: Yunnan: Lancan county, holotype: male, in coll. NEFU.
  *Distribution*. China (Yunnan) [7,13].
7. ***Evonima maculata* Holloway, 2003**
  *Evonima maculata* Holloway, 2003: 32. Type locality: Borneo: Sarawak: Gunong Mulu Nat, Park, holotype: male, in coll. NHMUK.
  *Distribution*. Borneo (Brunei, Sabah, Sarawak) [8].
8. ***Evonima mandschuriana* (Oberthür, 1880)**
  *Erastria mandschuriana* Oberthür, 1880: 83. Type locality: [Far East Russian]: [Primorsky Krai]: Askold, holotype: male, in coll. NHMUK.
  *Distributions*. China, Far East Russia, Japan, Korea, Mongolia [7,8,9,13,14,21,22,23].
9. ***Evonima minora* (Eecke, 1926)**
  *Poecilonola minora* Eecke, 1926: 41. Type locality: [Indonesia] Sumatra: Fort de Kock, syntypes: 3 males, 1 female, in coll. RMNH.
  *Distribution*. Indonesia (Sumatra) [7,8,14,19].
10. ***Evonima ochritincta* (Hampson, 1901)**
  *Poecilonola ochrintincta* Hampson, 1901: 178. Type locality: [Sri Lanka] Ceylon: Haputale, holotype: male, in coll. NHMUK.
  *Distributions*. China (Taiwan), India (Mizoram), Indonesia, Sri Lanka, Thailand (Chiang Mai) [7,8,10,11,14,15,20,24].
11. ***Evonima palawanensis* Cha sp. nov.**
  *Distribution*. The Philippines (Palawan).
12. ***Evonima plagiola* (Hampson, 1898)**
  *Selca plagiola* Hampson, 1898: 441. Type locality: [Sri Lanka] Ceylon: Puttalam, holotype: male, in coll. NHMUK.
  *Distribution*. Sri Lanka [7,8,14,25].
13. ***Evonima ronkaygabori* Han & Hu, 2019**
  *Evonima ronkaygabori* Han & Hu, 2019: 442. Type locality: China: Yunnan: Jiangcheng holotype: male, in coll. NEFU.
  *Distributions*. China (Yunnan), India (Mizoram, West Bengal) [11,13].
14. ***Evonima shajiamaensis* Han & Hu, 2019**
  *Evonima shajiamaensis* Han & Hu, 2019: 440. Type locality: China: Yunnan: Weixi, Shajima bridge, holotype: male, in coll. SWUST.
  *Distribution*. China (Shaanxi, Sichuan, Yunnan) [7,13].
15. ***Evonima sinonanlinga* Hu, László, Ronkay & Wang, 2013**
  *Evonima sinonanlinga* Hu, László, Ronkay & Wang, 2013: 599. Type locality: China: Guangdong: Nanling, holotype: male, in coll. SCAU.
  *Distribution*. China (Guangdong, Shaanxi, Sichuan) [7,13,14].
16. ***Evonima tianmuensis* Hu, Yu & Huang, 2020**
  *Evonima tianmuensis* Hu, Yu & Huang, 2020: 291. Type locality: China: Zhejiang: Mt. Tianmushan, holotype: male, in coll. Zhi-Peng Liao.
  *Distribution*. China (Zhejiang) [7].
17. ***Evonima unicolor* László, Ronkay & Witt, 2010**
  *Evonima unicolor* László, Ronkay & Witt, 2010: 30. Type locality: N. Thailand: Nan, 5 km N of Ban Luang, between Pi Nai and Pi Tai, holotype: male, in coll. MWM.
  *Distribution*. Thailand (Nan) [7,10,12,14].
18. ***Evonima westafricana* Hacker, 2012**
  *Evonima westafricana* Hacker, 2012: 218. Type locality: [Côte d’Ivoire] Ivory Coast: Man, holotype: male, in coll. ZSM.
  *Distributions.* Côte d’Ivoire, Gabon, Ghana, Liberia, Nigeria, Sierra Leone [6,18].
19. ***Evonima xanthoplaga* (Hampson, 1911)**
  *Roeselia xanthoplaga* Hampson, 1911: 399. Type locality: [India]: Sikhim [Sikkim], holotype: female, in coll. NHMUK.
  *Distributions*. China (Guizhou, Yunnan), India (Arunachal Pradesh, Sikkim), Thailand (Chiang Mai, Nan) [7,8,11,12,13,20].

## 4. Discussion

Recent research has uncovered many new species and genera, contributing to Nolidae taxonomy. Most studies have focused on the Oriental region, and this study also contributes to exploring Nolidae diversity. Palawan Island remains underexplored, with only a few moths and some important butterflies documented (City ENRO, pers. comm., 2022). Roughly, over 500 species of butterflies and only around 500 species of moths have been recorded from Palawan Island [26,27,28,29,30,31,32,33]. This study broadens taxonomic knowledge, but the present study is limited to northern Palawan.

Both *Wittonola* László, Ronkay & Ronkay, 2015 and *Aeneanola* László, Ronkay & Ronkay, 2013 are small genera restricted to their type localities, except for *A. acontioides* (Walker, 1862), the only *Aeneanola* species recorded in the Philippines. *Evonima* Walker, 1862 consists of over 15 species, with only *E. albifurca* (Hampson, 1914) previously described from the Philippines. This study marks the first record of *Wittonola* from the Philippines and of *Aeneanola* and *Evonima* from Palawan—each represented by new species.

Although this research relies on morphological characteristics, molecular analysis is essential for further validation. Genetic studies could clarify phylogenetic relationships, and the three newly described species provide valuable material for future research. Expanding surveys to other regions and integrating DNA-based analyses will improve Nolidae taxonomy and our understanding of its phylogenetic relationships.

## Data Availability

The original contributions presented in this study are included in the article. Further inquiries can be directed to the corresponding author.

## Abbreviations

The following abbreviations are used in this manuscript:

MWM/ZSM	Bavarian State Collection of Zoology, (Zoologische Staatssammlung München), Münich, Germany
NHMUK	Natural History Museum, London, United Kingdom
OUMNH	Oxford University Museum of Natural History, Oxford, United Kingdom
RMNH	Royal Museum of Natural History (Naturalis Biodiversity Center), Leiden, the Netherlands
SCAU	South China Agricultural University, Guangzhou, China
SWUST	Southwest University of Science and Technology, Minyang, China
